# Clinical, Neuroimaging and Video Electroencephalography Findings in Children With Congenital Zika Syndrome: An Analysis From a Neurorehabilitation Centre

**DOI:** 10.1002/jdn.70115

**Published:** 2026-03-16

**Authors:** Adilina Soares Romeiro Rodrigues, Pedro Ykaro Fialho Silva, Ramiro Pinheiro Rodrigues, Kylvia Giselle Fernandes Dantas Pinho, Sthefani da Costa Penha, Álvaro Jorge Madeiro Leite

**Affiliations:** ^1^ Graduate Program in Public Health Federal University of Ceará Fortaleza CE Brazil; ^2^ Sarah Network of Rehabilitation Hospitals Fortaleza CE Brazil

**Keywords:** child development, epidemiology, microcephaly, neuroimaging, video electroencephalogram, Zika virus

## Abstract

**Introduction:**

Congenital Zika syndrome (CZS) represents a spectrum of fetal and neonatal abnormalities resulting from in utero Zika virus (ZIKV) transmission during pregnancy. Given the severe multisystem disabilities, relative recency of the epidemic and limited long‐term data, comprehensive characterization at specialized centres is crucial.

**Objective:**

This study aimed to examine clinical symptoms, brain imaging and brain activity (video electroencephalography, VEEG) patterns in children with CZS receiving care at a specialized rehabilitation centre.

**Method:**

We conducted a cross‐sectional study from August 2018 to January 2019 with 48 children diagnosed with CZS according to the Brazilian Ministry of Health criteria. We collected clinical data from electronic medical records.

**Results:**

The most common clinical problems included bladder and bowel incontinence (97.9%), epilepsy (85.5%), facial abnormalities (89%), swallowing difficulties (83.3%), excessive irritability (81.3%), eye misalignment (75%), sleep problems (72.9%), acid reflux (62.0%) and vision problems (62.5%). Brain imaging revealed reduced brain tissue volume (95.8%), abnormal corpus callosum (91.1%), enlarged fluid‐filled spaces in the brain (89.5%), calcium deposits at the brain's outer layers (78.3%) and abnormally thick brain folds (71.1%). We found significant links between bone/muscle malformations and both white matter disease (*p* = 0.036) and enlarged brain ventricles (*p* = 0.031).

**Conclusion:**

Children with CZS consistently show motor difficulties, multiple clinical problems and characteristic brain abnormalities. These findings predict significant limitations in daily activities, movement and cognitive–social development.

## Graphical abstract

Children with congenital Zika syndrome followed at a neurorehabilitation centre demonstrated severe neurological impairment characterized by microcephaly, cortical malformations, intracranial calcifications and multifocal epileptiform activity. Integrated clinical, neuroimaging and video‐electroencephalographic (EEG) findings highlight the extensive neurodevelopmental impact and need for long‐term multidisciplinary care.

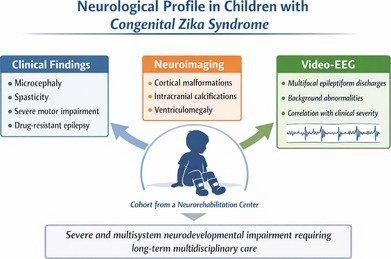

## 1 Introduction

Congenital Zika syndrome (CZS) represents a spectrum of fetal and neonatal abnormalities resulting from in utero Zika virus (ZIKV) transmission during pregnancy. The syndrome is characterized by severe microcephaly with skull collapse, ocular damage, arthrogryposis, hypertonia and multisystem involvement (Mattos et al. 2023; Daza et al. 2021). Affected children commonly present with cerebral parenchymal calcifications, ventriculomegaly, cortical malformations (including lissencephaly and pachygyria), corpus callosum dysgenesis and posterior fossa abnormalities (Mattos et al. 2023; Daza et al. 2021; Martins et al. 2025). Ophthalmologic and auditory impairments contribute substantially to long‐term disability, and importantly, the clinical spectrum extends beyond isolated microcephaly, with a significant proportion of exposed children without microcephaly still demonstrating neurodevelopmental abnormalities (Freitas et al. 2020).

The neuroimaging signature of CZS is characterized by a distinctive triad: parenchymal intracranial calcifications, malformations of cortical development and ventriculomegaly (Daza et al. 2021; Martins et al. 2025). Unlike other congenital infections, calcifications in CZS are typically cortico‐subcortical rather than periventricular (Freitas et al. 2020). Quantitative imaging markers, including Evans' index, cortical thickness and composite severity scores, demonstrate strong associations with neurodevelopmental outcomes and serve as prognostic tools (Alves et al. 2021; Leão et al. 2020; Esper et al. 2022). The timing of maternal infection influences severity, with first‐trimester ZIKV infection associated with more severe brain abnormalities and microcephaly (Mendes et al. 2020).

Following the 2015–2016 outbreak, large prospective cohorts have documented substantial morbidity among exposed infants. One major cohort reported 24.2% congenital microcephaly among 296 confirmed exposures, with high frequencies of multisystem abnormalities in both microcephalic and nonmicrocephalic infants (Freitas et al. 2020) Regional studies indicate approximately 20% of exposed children develop adverse CZS‐compatible outcomes (Marques, Amarante, et al. 2023). Despite the COVID‐19 pandemic shifting global health priorities, ongoing cohort follow‐up demonstrates persistent severe disability, supporting continued clinical surveillance (Martins et al. 2025; Freitas et al. 2020; Leão et al. 2020).

Systematic characterization of clinical, neuroimaging and electroencephalographic (EEG) findings is essential for diagnosis, prognostication, and intervention planning. Quantitative imaging indices predict delays across cognitive, language and motor domains, enabling risk stratification for early intervention (Leão et al. 2020; Esper et al. 2022). Video electroencephalography (VEEG) provides critical prognostic information, as continuous epileptiform discharges (CEDs) are associated with severe microcephaly, earlier seizure onset, drug‐resistant epilepsy and worse developmental outcomes (Campos et al. 2024; Corrêa et al. 2021). Early phenotyping through imaging and EEG enables prioritization for habilitation, seizure control, visual and hearing rehabilitation and family support services (Campos et al. 2024; Esper et al. 2020; de Souza et al. 2022).

Given the severe multisystem disabilities, relative recency of the epidemic and limited long‐term data, comprehensive characterization at specialized centres is crucial. Understanding specific clinical, neuroimaging and VEEG profiles contributes to timely diagnosis, evidence‐based interventions and longitudinal follow‐up protocols. Therefore, this study aimed to describe the clinical, neuroimaging and VEEG findings in children with CZS attending a specialized neurorehabilitation centre.

## 2 Methods

### 2.1 Study Design and Setting

This was a cross‐sectional, descriptive study conducted at the Neurorehabilitation Center of the Sarah Network of Rehabilitation Hospitals, Fortaleza, Ceará, Brazil, from August 2018 to January 2019.

### 2.2 Sample Selection

The sample consisted of children aged 12–48 months with a confirmed diagnosis of CZS, who were followed at a specialized outpatient clinic. All cases had a positive and reactive result for Zika virus in biological samples collected from the newborn up to the eighth day of life. Additionally, according to the diagnostic protocol in effect at the time, all children had negative or inconclusive results for at least one of the STORCH infections (syphilis, toxoplasmosis, rubella, cytomegalovirus and herpes simplex), thereby reducing the likelihood of other congenital infections interfering with the clinical and neurological findings.

The children included in the study had been reported at birth to the Brazilian Ministry of Health by the respective State Health Secretariats, and the diagnosis of CZS was confirmed based on clinical, laboratory and radiological criteria established in the integrated surveillance and healthcare guidelines implemented during the National Public Health Emergency of National Importance.

**FIGURE 1:**
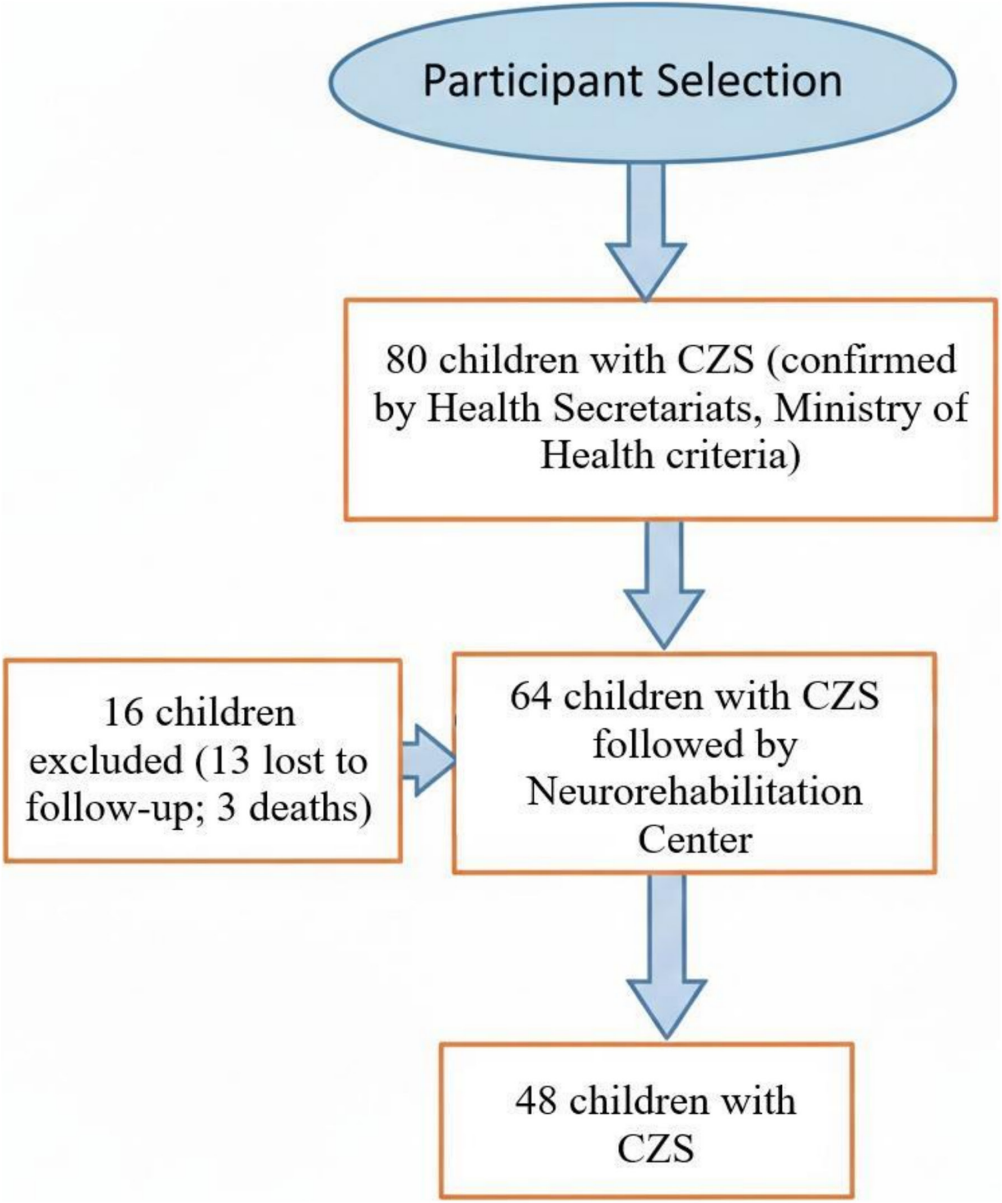
Participant selection.

Exclusion criteria comprised failure to attend follow‐up appointments, lack of authorization from a parent or legal guardian and the presence of microcephaly or brain malformations attributable to causes other than congenital Zika virus infection. The participant selection process is illustrated in Figure 1.

### 2.3 Data Collection

Data were collected from electronic medical records and through interviews using a semistructured instrument. This instrument covered socioeconomic data including maternal age and education, family origin, residence area, receipt of continuous cash benefit (Benefício de Prestação Continuada da Assistência Social—BPC) and sanitation conditions. Gestational data included prenatal care, signs and symptoms related to ZIKV infection, trimester of symptom occurrence, type of delivery, clinical complications, medication use, congenital infections and confirmatory exams.

Clinical data encompassed diagnosis and classification of cerebral palsy, phenotypic changes such as ear implantation, wide‐set eyes, flat face, craniofacial disproportion, microphthalmia (small eyes), retrognathia (receding jaw), hypotelorism (close‐set eyes) and scalp skin redundancy. We also documented alterations including epilepsy, spasticity, postural changes, exaggerated primitive reflexes, hyperexcitability, orthopaedic deformities, visual and auditory impairment, abnormal eye movements, neurogenic irritability, symptomatic gastroesophageal reflux, swallowing difficulties, intestinal constipation, sphincter incontinence, surgeries and neurodevelopmental delay assessed using pediatric developmental milestones.

Perinatal data included gestational age, weight, height, head circumference at birth and Apgar score (a newborn health assessment). Neurological exams included neuroimaging results from MRI (Magnetic Resonance Imaging) and CT (Computed Tomography) scans, as well as VEEG (video electroencephalography) results.

Motor acquisitions were assessed using the Pan‐American Health Organization (PAHO) guidelines for child development surveillance (Alves et al. 2021). The failure to meet a single milestone was considered a risk for developmental delay, necessitating the earliest possible intervention. Anthropometry measurements including weight, height and head circumference were measured according to the Surveillance of Food and Nutrition (SISVAN) and Child and Adolescent Nutritional Assessment manuals (Leão et al. 2020; Esper et al. 2022). Apgar scores were analysed, noting that according to the WHO, an Apgar score less than 7 at 5 min indicates severe birth asphyxia and identifies a high‐risk newborn (Mendes et al. 2020).

#### 2.3.1 Neuroimaging and Video Electroencephalography Analysis

Neuroimaging scans were analysed by the imaging diagnostic team at the Neurorehabilitation Center. Magnetic Resonance Imaging (MRI) was performed on a GE Signa HDx 1.5 T equipment, and Computed Tomography (CT) on a Brilliance CT 64‐DS scanner.

VEEG recordings were performed by the Neurorehabilitation Center team using a NEUROTEC (Neuromap) digital recorder with a sensitivity of 7 μV/mm, low‐frequency filters of 0.6 Hz, high‐frequency filters of 70 Hz and a speed of 1.5 cm/s. Electrodes were placed according to a modified 10–20 system for newborns, using a bipolar montage (Fp1‐C3, C3‐01, Fp1‐T3, T3‐01, Fp2‐C4, C4‐02, Fp2‐T4, T4‐02 and Cz‐Oz). All patients were recorded during spontaneous sleep for a duration of 20–30 min.

### 2.4 Statistical Analysis

All data were collected, stored and analysed using the REDCap electronic tool implemented at the University Hospital of the Federal University of Ceará. Categorical variables were described using absolute values and percentages. Numerical variables were described using mean, median and standard deviation. Associations between variables were analysed using frequency and prevalence rate. Statistical analyses were performed using the Jamovi 1.0 software and Microsoft Excel 2016.

### 2.5 Ethical Considerations

The research was approved by the Ethics and Research Committee of the Neurorehabilitation Center (CAAE 90324318.6.3001.5054), adhering to Resolution 466/2012 of the National Health Council. Informed consent was obtained from the legal guardians of the children.

## 3 Results

A total of 80 children with confirmed ZIKV infection, according to municipal and state health secretariats and Ministry of Health criteria, were initially selected for the study. Of these, 48 children met inclusion criteria and completed the study. The mean age of the children was 33 months, and 22 (46.8%) were male.

The mean Apgar score was 8.1 ± 1.4 at the first minute and 9.0 ± 0.5 at the fifth minute. All children met the clinical criteria for cerebral palsy and were classified as having spastic tetraplegia. According to the Gross Motor Function Classification System (GMFCS), the majority were classified as level V, and 47 (97.9%) were spastic.

The mean head circumference at birth was 29.5 cm, consistent with severe microcephaly (< 3rd percentile for gestational age and sex). The demographic and perinatal characteristics of the participants are summarized in Table 1.

**TABLE 1:** Demographic and perinatal characteristics data of children with congenital Zika syndrome treated at the Sarah Neurorehabilitation Center. Fortaleza, CE, Brazil, 2019.

Variables	*n*	%
Age (months)	33.04 ± 3.81	
GA (at birth)	38.11 ± 2.87	
Sex		
Female	25	53.2
Male	22	46.8
Race		
Mixed‐race	26	54.1
White	20	41.7
Black	2	4.2
APGAR (mean + SD)		
First minute	8.1 + 1.4	
Fifth minute	9.0 + 0.5	

Most clinical alterations investigated had a high prevalence and included vesicointestinal (bladder and bowel) incontinence in 97.9% of children, epilepsy in 85.5%, dysmorphism (facial abnormalities) in 89%, swallowing difficulties in 83.3%, neurogenic irritability in 81.3%, strabismus (eye misalignment) in 75%, sleep disturbance in 72.9%, gastroesophageal reflux in 62.0% and visual impairments in 62.5%. Lower prevalence conditions included hip dislocation (41.7%), kyphosis (spine curvature, 41.7%), bruxism (teeth grinding, 35.4%), cardiac alterations (22.9%), arthrogryposis (joint contractures, 8.3%) and congenital clubfoot (6.3%). The most frequent primitive reflexes observed were Babinski (58.3%), where toes fan upward when the foot sole is stroked and positive support (50.0%), where legs stiffen when feet touch a surface. The distribution of these clinical findings is summarized in Table 2.

**TABLE 2:** Clinical data of children with congenital Zika syndrome treated at the Sarah Neurorehabilitation Center. Fortaleza, CE, Brazil, 2019.

Cerebral palsy diagnosis	48	100
Topography and motor alterations		
Spastic tetraplegia	47	97.9
Mixed tetraplegia	1	2.1
Spasticity	47	97.9
Faecal incontinence	47	97.9
Urinary incontinence	47	97.9
Epilepsy	41	85.5
Dysmorphism	43	89.6
Use of anticonvulsants	41	85.4
Dysphagia	40	83.3
Neurogenic irritability	39	81.3
Sleep disturbances	35	72.9
Visual impairment	30	62.5
Pneumonia	30	62.5
Gastroesophageal reflux	30	62.5
Sialorrhea	30	62.5
Nystagmus	27	56.3
Kyphosis	20	41.7
Hip subluxation	20	41.7
Bruxism	17	35.4
Subluxation	15	31.3
Cardiac abnormalities	11	22.9
Auditory neurosensory abnormality	11	22.9
Arthrogryposis	4	8.3
Congenital clubfoot	3	6.3

Regarding VEEG findings, 21 (45.7%) children showed signs suggestive of structural brain tissue damage, diffusely localized in 24 (61.5%). Epileptiform activity was observed in 41 (85.5%) children, with multifocal activity (occurring in multiple brain regions) in 24 (58%) of them.

The most prevalent neuroimaging findings were corpus callosum abnormalities (91.1%), calcification at the cortico‐subcortical junction (78.3%), cortical abnormality such as pachygyria (thick brain folds, 71.1%), leukodystrophy (white matter disease, 56.5%), mild ventriculomegaly (16.3%), severe ventriculomegaly (enlarged brain ventricles, 47.6%) and moderate ventriculomegaly (35.7%), basal ganglia calcification (41.3%) and moderate to severe reduction in cerebral parenchyma (brain tissue) volume (41.3%).

## 4 Discussion

### 4.1 Head Circumference and Neurodevelopmental Outcomes

The mean head circumference at birth in our sample was 29.51 cm. Smaller head circumference at birth, even within the normocephalic range, has been associated with lower cognitive and language scores in longitudinal follow‐up of antenatally exposed children, with head circumference *z*‐scores correlating significantly with neurodevelopmental outcomes in adjusted analyses (Mattos et al. 2023; Mulkey 2020).

Furthermore, ventriculomegaly on neonatal imaging adds independent predictive value beyond head circumference alone, with increases in Evans' index (reflecting ventricular enlargement) independently predicting delays across receptive and expressive language, cognitive, gross motor and fine motor domains on Bayley‐III assessments even after adjusting for head circumference (Daza et al. 2021). The timing of maternal infection also influences microcephaly severity, as first‐trimester ZIKV infection has been associated with more severe brain CT abnormalities and lower head circumference *z*‐scores at birth compared to later gestational exposure (Martins et al. 2025; Mendes et al. 2020). In our studied sample, severe microcephaly was defined as a percentile less than 3, with a mean age of 33.04 ± 3.81 months and a mean gestational age of 39.6 ± 1.9 weeks, consistent with the severe end of the CZS spectrum described in recent literature.

### 4.2 Sex Distribution

Regarding sex distribution, 50% of the children in our sample were male. Reported sex ratios in CZS cohorts vary across the literature, with some series showing approximately equal proportions of males and females among confirmed antenatally exposed infants, while certain clinic cohorts have noted a male predominance (Mattos et al. 2023; Freitas et al. 2020). While it has been suggested that male embryos may be more prone to congenital malformations due to chromosomal genetic differences, there is insufficient consistent mechanistic or longitudinal evidence in recent literature to definitively assert a true male predominance in CZS beyond observed cohort proportions (Paixão et al. 2022; Pereira‐Carvalho et al. 2023).

### 4.3 Cerebral Palsy Prevalence and Motor Outcomes

Cerebral palsy was diagnosed in 100% of the children in our studied sample. This finding is consistent with recent cohort studies demonstrating that motor impairment in CZS is commonly severe, with the majority of affected children classified at high Gross Motor Function Classification System (GMFCS) levels (Melo et al. 2020). Recent longitudinal cohorts have reported that 81% of children with CZS were GMFCS level V, and more than 80% had spastic tetraparesis or equivalent severe neuromotor phenotypes (Freitas et al. 2020; da Costa et al. 2024).

The high prevalence in our sample, compared to an earlier 2021 study reporting 89.7% (Takahasi et al. 2021), likely reflects our specialized rehabilitation centre setting, which serves children with more severe neurological impairment. Severe malformations of cortical development on neuroimaging and smaller head circumference at birth have been statistically associated with worse gross motor function and lower Gross Motor Function Measure (GMFM) scores (da Costa et al. 2024).

**FIGURE 2:**
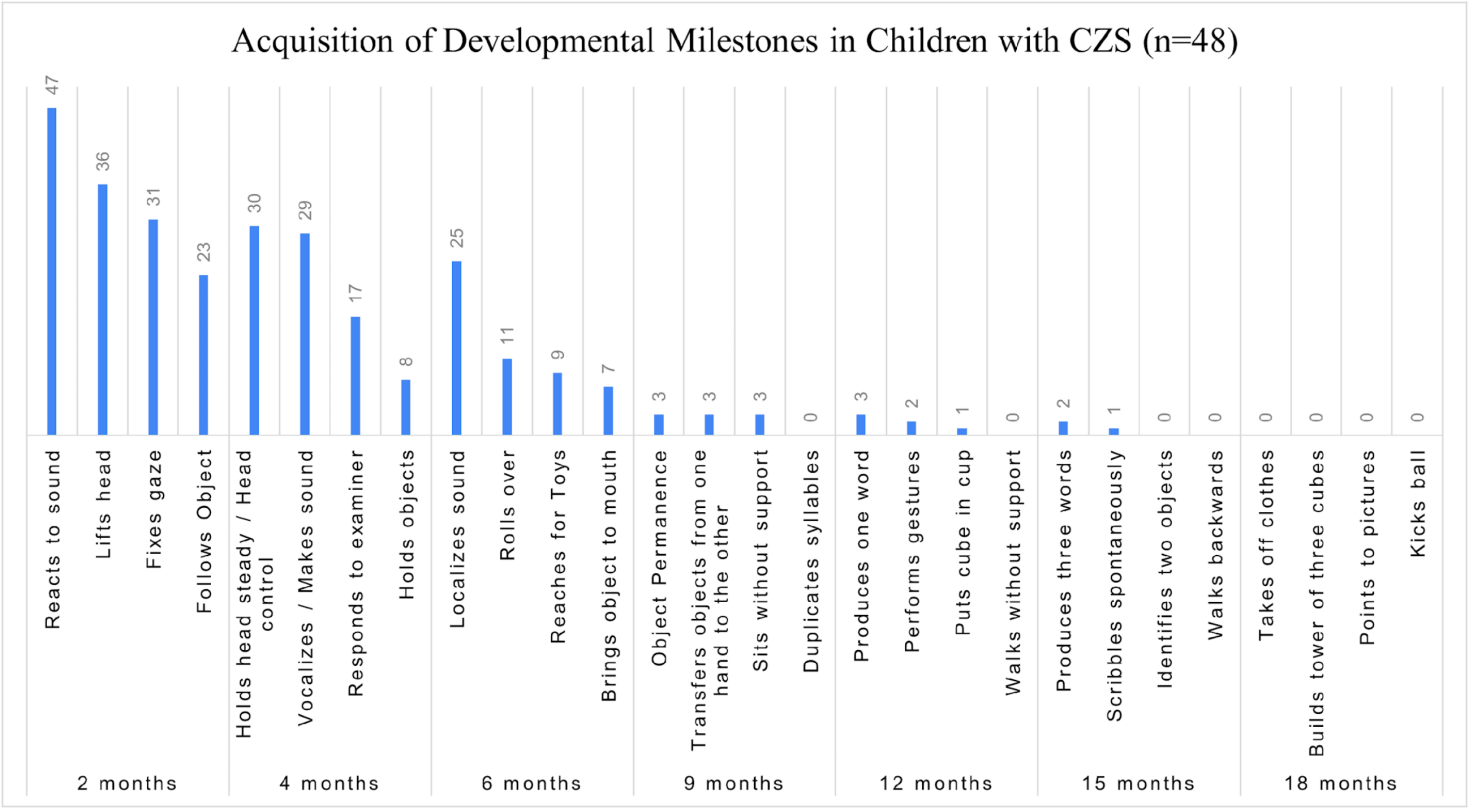
Developmental milestones according to expected age in children with congenital Zika virus syndrome, treated at the Sarah Neurorehabilitation Center. Fortaleza, CE, Brazil, 2019.

ICF‐based assessments of preschool‐aged children with CZS have reported very high rates of severe impairment across cognitive, motor tone and speech domains, with pervasive limitations in transfers and mobility (de Souza et al. 2022; Marques, Carvalho, et al. 2023). The distribution of developmental milestones according to expected age in the studied children is presented in Figure 2.

### 4.4 Epilepsy and Video Electroencephalography Findings

In our VEEG findings, epileptiform activity was present in 85.5% of children, consistent with the high frequency of epilepsy documented in recent CZS cohorts (Maia et al. 2021). Longitudinal studies have reported epilepsy in approximately 64%–71% of children with CZS, with infantile spasms predominant in the first year of life and focal seizures becoming more frequent in the second year (Mdcg et al. 2020). Recent prospective EEG studies have identified CEDs in approximately 40% of children with CZS, frequently bilateral in distribution (van der Linden et al. 2020). Importantly, CEDs have been strongly associated with severe congenital microcephaly, earlier seizure onset (before 6 months of age), more severe CT lesion burden and significantly worse neurodevelopmental outcomes (Campos et al. 2024). The presence of CEDs has also been linked to drug‐resistant epilepsy, with separate follow‐up studies documenting substantial proportions of children with pharmacoresistant seizures and West syndrome phenotypes requiring complex management strategies (Cavalcante et al. 2022). The main VEEG findings observed in the study population are presented in Table 3.

**TABLE 3:** Video EEG findings of children with congenital Zika syndrome treated at the Sarah Neurorehabilitation Center. Fortaleza, CE, Brazil, 2019.

Variables	*n*	%
Age‐appropriate maturation signs		
Mild	11	23.9
Mild/moderate	5	10.9
Moderate	20	43.5
Moderate/severe	4	8.7
Severe	6	13.0
Signs suggestive of structural injury		
Absent	19	41.3
Present	21	45.7
Not identified	6	13.0
Location of these signs		
Localized	15	38.5
Diffuse	24	61.5
Presence of epileptiform activity	41	85.5
Types of epileptiform activity		
Focal	15	36.6
Multifocal	24	58.5
Generalized	2	4.9

### 4.5 Primitive Reflexes, Postural Reactions and Motor Development

The persistence of primitive reflexes in our sample reflects neurological immaturity and severe brain injury. None of the children in our cohort achieved a functional form of locomotion, which directly impacts the acquisition of developmental milestones and independence in daily living activities. No child reached the expected milestone for their age, reflecting significant neurodevelopmental delay. The distribution of primitive reflexes observed in the participants is presented in Table 4.

**TABLE 4:** Primitive reflexes (*n* = 48).

Variables	*n*	%
Primitive reflexes		
ATNR	10	20.8
Moro	21	43.8
Positive support	24	50.0
Placing	6	12.5
Babinski	28	58.3
Automatic walking	4	8.3

The highest number of achieved milestones was observed in the 2‐ to 4‐month age range, consistent with early developmental arrest. We observed a direct correlation between increased muscle tone and persistence of primitive reflexes and an inverse correlation between tone and postural reactions. Increased muscle tone contributes to functional deficits in the upper and lower limbs, lack of skill acquisition and poor motor performance (de Souza et al. 2022).

The severe motor impairment observed in our cohort, with all children classified as having cerebral palsy and the majority at GMFCS level V, is consistent with recent literature demonstrating that severe cortical malformations predict worse motor outcomes and that early rehabilitation is essential despite the severity of impairment (Aguilar‐Ticona et al. 2021; Schuler‐Faccini et al. 2022; Pereira et al. 2020). Motor function and developmental status of the participants are summarized in Table 5.

**TABLE 5:** —Motor function and developmental status (*n* = 48).

Motor acquisitions		
Cervical control—Sitting		
Poor	21	43.8
Fair	16	33.3
Good	8	16.7
Absent	3	6.3
Prone		
Poor	22	45.8
Fair	16	33.3
Good	9	18.8
Absent	1	2.1
Supine		
Poor	11	22.9
Fair	11	22.9
Good	22	45.8
Absent	4	8.3
Trunk balance		
Poor	33	68.8
Fair	12	25.0
Good	3	6.3
Rolling		
Partial	9	18.8
Total	6	12.5
Does not roll	33	68.8
GMFCS		
II	1	2.1
IV	3	6.3
V	43	89.6

### 4.6 Neuroimaging Findings

**FIGURE 3:**
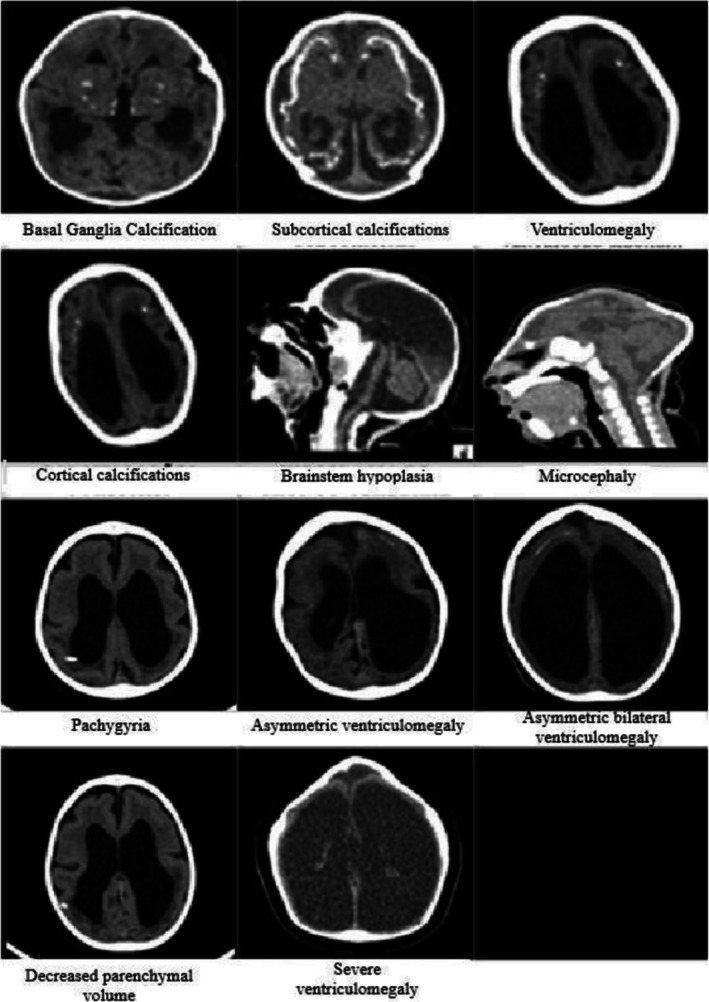
Alteration findings in head computed tomography of children with congenital Zika virus syndrome, treated at the Sarah Neurorehabilitation Center. Fortaleza, CE, Brazil, 2019.

In our neuroimaging findings, 100% of the children presented with ventriculomegaly, ranging from mild to severe. This is consistent with recent cohort studies reporting ventriculomegaly in 86%–100% of cases (de Fatima Vasco Aragao et al. 2016; Christoff et al. 2023). Ventriculomegaly is a frequent and characteristic imaging finding in CZS, and when quantified using measures such as Evans' index or radiologic severity scores, it correlates strongly with poorer neurodevelopmental outcomes and lower head circumference measures (Christoff et al. 2023). Greater ventricular dilation implies greater neuronal loss and represents extensive cerebral damage. When associated with severe microcephaly, ventriculomegaly significantly impacts neurodevelopment and can lead to spasticity, postural changes and persistence of primitive reflexes, resulting in a poor prognosis for daily living activities (Carvalho et al. 2019). The main alterations observed on head computed tomography are presented in Figure 3.

### 4.7 Characteristic Neuroimaging Features

Corpus callosum abnormalities constitute a characteristic finding of CZS, observed in the majority of affected children (Carvalho et al. 2019; Pool et al. 2019). Intracranial parenchymal calcifications represent another hallmark feature, with prevalence rates reaching 97.6% in cohorts of children with CZS‐associated cerebral palsy (Santos et al. 2024). Calcifications at the cortico‐subcortical junction, observed in approximately 78%–80% of cases, represent a distinctive feature differentiating CZS from other congenital infections, which typically present with periventricular rather than parenchymal calcifications (Daza et al. 2021; Pool et al. 2019). The neuroimaging findings observed in the study population are summarized in Table 6.

**TABLE 6:** Neuroimaging findings in children with congenital Zika syndrome treated at the Sarah Neurorehabilitation Center. Fortaleza, CE, Brazil, 2019.

Variables	*n*	%
Leukodystrophy		
Present	26	56.5
Absent	17	37.0
Not found	3	6.5
Volumetric reduction of brain parenchyma		
Mild	8	17.4
Moderate	19	41.3
Severe	19	41.3
Cortical abnormalities		
Lissencephaly	8	16.7
Polymicrogyria	2	4.2
Pachygyria	37	77.1
NA	5	10.4
Nonspecific sulcal/gyral abnormalities	—	—
Corpus callosum abnormalities	41	91.1
Ventriculomegaly		
Mild	7	16.3
Moderate	15	34.9
Severe	21	48.8
Ventricular septations	1	2.2
Cerebellar abnormalities (hemispheric hypoplasia)	5	10.9
Cerebellar abnormalities (vermis hypoplasia)	3	6.5
Brainstem hypoplasia or atrophy	12	26.7
Periventricular calcifications	9	19.6
Cortical calcifications	8	17.4
Corticossubcortical junction calcifications	36	78.3
Basal nuclei calcifications	19	41.3
Brainstem calcifications	—	—
Cerebellar calcifications	—	—

### 4.8 Cortical Malformations and Clinical Significance

Malformations of cortical development, including pachygyria and agyria (lissencephaly), occur in 80%–100% of severely affected cases and are most strongly linked to first‐trimester maternal infection (Daza et al. 2021; Santana et al. 2021). These cortical malformations result from interrupted neuronal migration during the first half of gestation and manifest clinically with drug‐resistant epilepsy, focal neurological deficits, delayed psychomotor development, intellectual disability, global hypotonia and impaired visuospatial and fine motor control (Carvalho et al. 2019). Semi‐automated MRI severity scores correlate strongly with Bayley and motor scores, validating neuroimaging as a prognostic tool for risk stratification and early intervention planning (Carvalho et al. 2020).

### 4.9 Visual Impairment and Ophthalmologic Findings

Visual impairment results from damage to ocular structures and cortical/subcortical brain areas, with structural ophthalmologic abnormalities in severely affected subgroups. Visual pathway injury includes occipital cortical volume loss, optic nerve and chiasmal atrophy and retinal scarring (Daza et al. 2021; Henderson et al. 2021). Cortical visual impairment explains many cases of poor vision despite relatively preserved ocular structures, significantly limiting interaction, exploration and learning capacity in children already facing severe motor and cognitive challenges (Pool et al. 2019). Comprehensive ophthalmologic evaluation and visual rehabilitation are essential components of multidisciplinary care.

### 4.10 Early Intervention and Rehabilitation

All children with CZS require early multidisciplinary intervention regardless of condition severity and prognosis (Mattos et al. 2023; Alves et al. 2021). Interventions include early stimulation, family involvement throughout rehabilitation and health education to prevent complications and promote quality of life and social inclusion. Early phenotyping through imaging and EEG enables prioritization for habilitation, seizure control, visual and hearing rehabilitation and family support services (Lage et al. 2019).

Coordinated long‐term surveillance and social support are essential given the high care needs that burden families and healthcare systems. The epidemic revealed structural fragility in public policies protecting vulnerable populations, with violations of rights observed in medical care access, delayed diagnosis, discrimination and harassment. Characterization studies enable improved quality of life through evidence‐based intervention planning and comprehensive multidisciplinary care, while informing resource planning and policies to support lifelong care needs.

### 4.11 Study Limitations

Regarding the study's limitations, the sample selection resulted in all children having severe neurological injury and neurodevelopmental impairment, thus limiting the generalizability of our findings across the full spectrum of CZS, which includes children with milder phenotypes and those exposed without microcephaly who may still have developmental abnormalities. Another limitation is that children with radiological findings suggestive of white matter abnormalities would be better evaluated by brain MRI rather than CT scans alone, as MRI provides superior tissue contrast and characterization of white matter, cortical malformations and posterior fossa structures. Future studies should include longitudinal MRI assessments and standardized neurodevelopmental testing across the full spectrum of CZS severity to better understand the range of outcomes and identify modifiable factors that may improve long‐term prognosis.

## 5 Conclusion

The current study demonstrated that children with CZS exhibit frequent motor and clinical alterations, as well as changes in neuroimaging and VEEG, which can influence mobility and socio‐cognitive functions. The motor and sensory dysfunctions interfere with the acquisition and performance of basic and complex milestones. Children with microcephaly have atypical neurodevelopment, limiting the acquisition of normal patterns of functional movements.

The alterations in the neurodevelopment of these children indicate a necessity for access to social policies, reference institutions and other specialized services. Determinant and conditioning factors such as food, housing, basic sanitation, family income, education, leisure and access to guaranteed benefits, essential services and transport can play a role in this process. It is relevant to understand the reality of the families to plan actions developed in the process of care, treatment and health promotion.

## Funding

The researcher used personal funds to support the research.

## Ethics Statement

The research project that forms part of this study was approved by the Ethics Committee of the Associação das Pioneiras Sociais—Sarah Network of Rehabilitation Hospitals (CAAE 90324318.6.3001.5054).

## Conflicts of Interest

The authors declare no conflicts of interest.

## Data Availability

Data sharing is not applicable to this article as no datasets were generated or analysed during the current study.
